# Supplementary material for: Bad-metal relaxation dynamics in a Fermi lattice gas
Source: Nat Commun. 2019 Apr 8;10:1588. doi: 10.1038/s41467-019-09526-x (PMC6453915; doi:10.1038/s41467-019-09526-x)
Supplement: Supplementary file 1 — Supplementary Information [file 41467_2019_9526_MOESM1_ESM.pdf]

Supplementary Information: Bad-Metal Relaxation Dynamics in a Fermi Lattice Gas

W. Xu,[1] W.R. McGehee,[2] W.N. Morong,[3] and B. DeMarco[3,*]

[1]*Department of Physics, Massachusetts Institute of Technology, Cambridge, MA, 02139, USA*

[2]*Center for Nanoscale Science and Technology, National Institute of Standards and Technology, Gaithersburg, Maryland 20899, USA*

[3]*Department of Physics, University of Illinois at Urbana-Champaign, Urbana, Illinois 61801, USA*

(Dated: March 9, 2019)

* bdemarco@illinois.edu

SUPPLEMENTARY NOTE 1: EFFECTIVE CHEMICAL POTENTIAL AND TEMPERATURE

We use a relatively simple approach that leverages the adiabatic theorem of quantum mechanics and the absence of inter-particle interactions in the initially spin-polarized gas to estimate an effective chemical potential $\tilde{\mu}$ and temperature $\tilde{T}$. These thermodynamic parameters are used to generate the semi-classical phase space distributions required by the weak scattering calculation and are needed to determine the density-weighted density. Our method involves calculating the occupation of single-particle eigenstates in the combined lattice and trap potential by assuming that the lattice turn-on satisfies the adiabatic theorem of quantum mechanics. These populations are then grouped and sorted by energy to form a distribution that is fit to a Fermi-Dirac (FD) profile.

Before turning on the optical lattice potential, the spin-polarized, non-interacting gas is trapped in a parabolic potential. The distribution of atoms in the single-particle eigenstates, characterized by three quantum numbers n_x, n_y, and n_z, is therefore well described by a FD function $f_{n_x,n_y,n_z} = 1/\left[e^{(\epsilon_{n_x,n_y,n_z}-\mu)/k_{\mathrm{B}}T} + 1\right]$, where μ is the chemical potential, k_{B} is Boltzmann's constant, and T is the temperature. Here, the directions x, y, and z correspond to the lattice axes. To determine the occupations f_{n_x,n_y,n_x} in the eigenstates for $n_i = 1...300$, we measure the number of atoms N and temperature of the gas using time-of-flight imaging. The chemical potential μ is calculated by enforcing the constraint $N = \sum_{n_x,n_y,n_z} f_{n_x,n_y,n_z}$. We have checked that including more states does not significantly affect our results. We treat the trap as spherically symmetric, so that the energies are $\epsilon_{n_x,n_y,n_z} = \hbar\tilde{\omega}\left(n_x + n_y + n_z\right)$, where $\tilde{\omega}$ is the geometric mean of the trap frequencies. We have also verified that taking into account the different trap frequencies does not affect the best estimate for $\tilde{\mu}$ and $\tilde{T}$.

The single-particle eigenstates in the combined lattice–trap potential are also characterized by three quantum numbers [1, 2]. The lattice is turned on over 100 ms, which is much slower than all other timescales: $\hbar/t \approx 0.5$ ms, $\hbar/U \approx 55$ μs, and $\hbar/E_{\mathrm{bg}} \approx 4$ μs (at $s = 7$), where E_{bg} is the bandgap. We therefore assume that the adiabatic theorem of quantum mechanics is satisfied, so that the distribution of particles in the lattice–trap eigenstates is unchanged, that is, $\tilde{f}_{n_x,n_y,n_z} = f_{nx,ny,nz}$. The corresponding eigenenergies are $\tilde{E}_{n_x,n_y,n_z} = \tilde{E}_{n_x} + \tilde{E}_{n_y} + \tilde{E}_{n_z}$,

with

$$\tilde{E}_i = \begin{cases} \frac{\bar{\Omega}}{4} a_i(\alpha), & i \text{ even} \\ \frac{\bar{\Omega}}{4} b_{i+1}(\alpha), & i \text{ odd} \end{cases} \tag{1}$$

in the lattice–trap potential, where $\bar{\Omega} = m\bar{\omega}^2 d^2/2$, $\bar{\omega}$ is the geometric mean of the trap frequencies with the lattice light present, d is the lattice spacing, $\alpha = 4t\bar{\Omega}$, and $a_i(\alpha)$ and $b_{i+1}(\alpha)$ are the Mathieu characteristic values [1, 2]. We group and order the populations by energy to form a distribution $\tilde{f}_{\tilde{\epsilon}_j} = \sum_{\tilde{E}_{n_x,n_y,n_z} = \tilde{\epsilon}_j} \tilde{f}_{n_x,n_y,n_z}$, where j indexes the ordered, unique energies. We fit the distribution of occupations $\tilde{f}_{\tilde{\epsilon}_j}$ to a FD function $1/\left[e^{(\tilde{\epsilon}_j - \tilde{\mu})/k_\mathrm{B}\tilde{T}} + 1\right]$ with an effective chemical potential $\tilde{\mu}$ and temperature $\tilde{T}$ as free parameters. The fit minimizes the sum of the squared difference at each $\tilde{\epsilon}_j$ between $\tilde{f}_{\tilde{\epsilon}_j}$ and the FD distribution.

Supplementary Figure 1 shows calculated $\tilde{f}_{\tilde{\epsilon}_j}$ and fitted FD distributions for $s = 4$, $N = 26,400$, and $T/T_\mathrm{F} = 0.22$, 0.44, and 1.16, which spans the full range of temperatures sampled in Fig. 3 in the main text. The results for the data in Fig. 5 of the Methods section for different s are similar to the lowest temperature point. The agreement between the computed $\tilde{f}_{\tilde{\epsilon}_j}$ and the fitted distributions are excellent, except for the highest temperature points. At the highest temperatures, the procedure we use generates a distribution that underestimates the occupancy for the lowest and highest energy states (Supplementary Figure 1c). As we will discuss, despite this issue we find that our procedure provides a density distribution that closely matches the experiment, and thus the failure apparent at high temperature in Supplementary Figure 1 likely cannot explain the discrepancy in Fig. 3 in the main text.

In Supplementary Figure 2, we show the predicted $\tilde{\mu}$ and $\tilde{T}$ for the conditions of Fig. 3 in the main text for different values of T/T_F before the lattice is turned on. The effective degeneracy is approximately conserved as the lattice is turned on (that is, $\tilde{T}/\tilde{T}_\mathrm{F} \approx T/T_\mathrm{F}$), and the effective chemical potential becomes negative for $T/T_\mathrm{F} \gtrsim 0.5$, as for a trapped gas.

To verify our approach, we compare predicted and measured density profiles for a spin-polarized gas in the lattice–trap potential. Comparing measured quasimomentum profiles is complicated by the failure of bandmapping at the edge of the Brilloun zone (BZ) [2]. To measure the density profile, we first transfer approximately 90% of the atoms to the $F = 7/2$ state using adiabatic rapid passage driven by a microwave-frequency magnetic field. This step is necessary to reduce the optical depth (OD) of the gas. We then image the gas in situ. The images are angularly averaged (along ellipses that match the aspect ratio

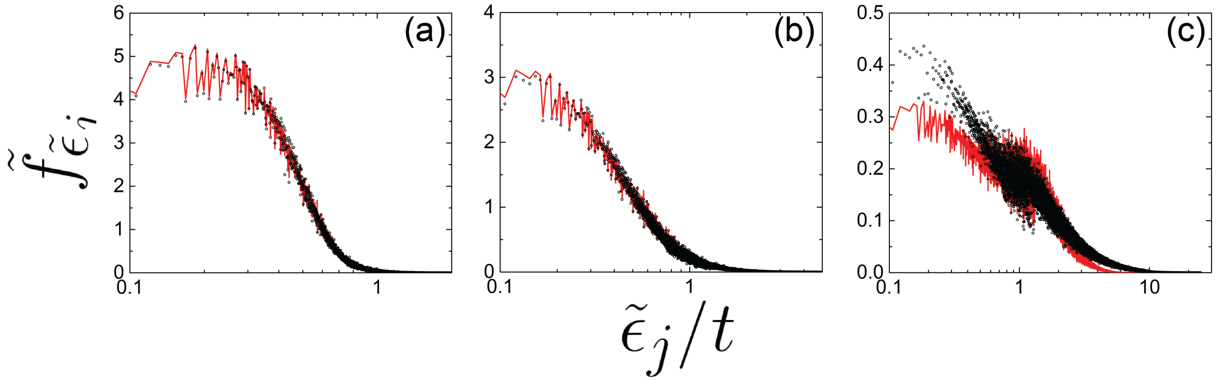

Supplementary Figure 1. Numerical data to determine effective chemical potentials and temperatures. The computed $\tilde{f}_{\tilde{\epsilon}_j}$ is shown for $s = 4$ and $T/T_{\rm F} = 0.22$ (a), 0.44 (b), and 1.16 (c) as open circles, and the fitted FD distribution is shown as a red line. A 20-point moving average filter is used to smooth the data, and approximately only one out of every 300 points are shown. Note the difference in vertical and horizontal scales.

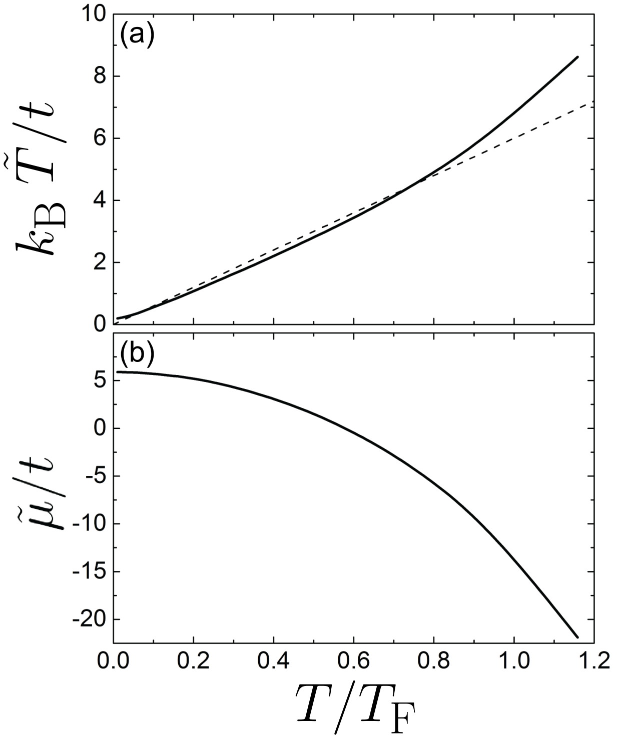

Supplementary Figure 2. The effective chemical potential and temperature scaled to the tunneling energy. The effective temperature (a) and chemical potential (b) are shown as solid lines. The dashed line marks the condition for $T/T_{\rm F}$ to remain unchanged when the lattice is superimposed on the gas, where $T_{\rm F} \approx 6t/k_{\rm B}$ in the lattice (according to a non-interacting calculation).

of the image) to produce a radial density profile. The measured radial profiles for $s = 4$ and $T/T_{\mathrm{F}} = 0.25$, 0.41, and 1.1 shown in Supplementary Figure 3 agree well with predicted profiles based on the estimated $\tilde{\mu}$ and $\tilde{T}$ and the semi-classical approximation. In the semi-classical approximation, the density $n(\vec{r}) = \int d^3\vec{q}\, \tilde{\rho}\left(\vec{r}, \vec{q}, \tilde{\mu}, \tilde{T}\right)/h^3$, where $\tilde{\rho}\left(\vec{r}, \vec{q}, \tilde{\mu}, \tilde{T}\right) = 1/\left[e^{\left(m\bar{\omega}^2 r^2/2 - 6t + 2t \sum_{j=x,y,z} \cos(\pi q_j/q_{\mathrm{B}}) - \tilde{\mu}\right)/k_{\mathrm{B}}\tilde{T}} + 1\right]$ is the semi-classical phase space distribution in the combined lattice–trap potential.

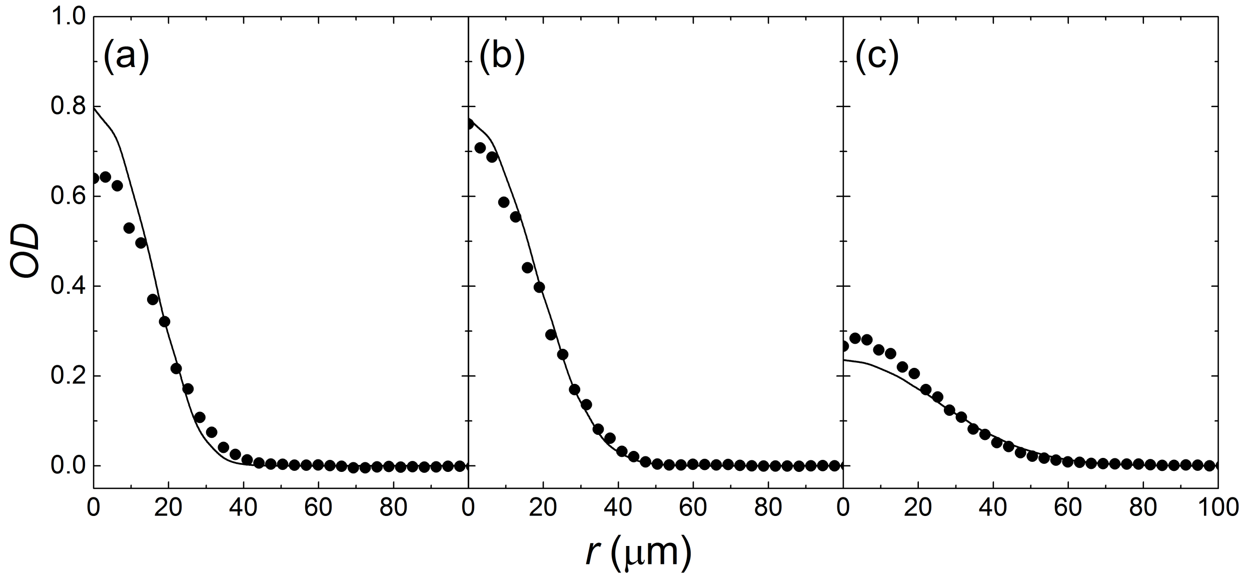

Supplementary Figure 3. Column-integrated radial density profiles for $T/T_{\mathrm{F}} = 0.25$ (a), 0.41 (b), and 1.1 (c) at $s = 4$ shown as the measured optical depth (OD) in the trap. The measured profiles (solid circles) are created from averages of 3–4 images, and the prediction (solid line) is based on $\tilde{\mu}$ and $\tilde{T}$. The predicted profile is scaled to match the number of atoms for each case: N=16500 (a), 22600 (b), and 15700 (c). In this figure, r is a scaled radius that follows contours of constant OD.

For the weak scattering calculation of the transport lifetime, we assume that the semi-classical phase space distribution in the lattice before the Raman pulse is a FD distribution $\xi\tilde{\rho}\left(\vec{r}, \vec{q}, \tilde{\mu}, \tilde{T}\right)$ in the $|\uparrow\rangle$ state, where $\xi = N/\left[\int d^3\vec{r} \int d^3\vec{q}\, \tilde{\rho}\left(\vec{r}, \vec{q}, \tilde{\mu}, \tilde{T}\right)/h^3\right]$ is a multiplicative factor used to fix the total atom number to the value in the experiment. This correction is necessary because the fitting procedure used to determine $\tilde{\mu}$ and $\tilde{T}$ is not constrained to correctly reproduce N. We approximate the Raman pulse as instantaneous, and use semi-classical distributions $\tilde{\rho}_{\uparrow}(\vec{r}, \vec{q}) = \xi(1-\gamma)\,\tilde{\rho}\left(\vec{r}, \vec{q}, \tilde{\mu}, \tilde{T}\right)$ and $\tilde{\rho}_{\downarrow}(\vec{r}, \vec{q}) = \xi\gamma\,\tilde{\rho}\left(\vec{r}, \vec{q} - \vec{\delta q}, \tilde{\mu}, \tilde{T}\right)$ for the relaxation rate calculation. Here, $\gamma \approx 0.35$ is the fraction of atoms transferred to the $|\downarrow\rangle$ state by the Raman pulse. The shifted momentum $\vec{q} - \vec{\delta q}$ of the $|\downarrow\rangle$ particles is re-mapped

to the first BZ by subtracting reciprocal lattice vectors if it exceeds the first BZ boundary. Time-of-flight measurements indicate that the Raman lasers do not transfer atoms out of the first BZ, which is expected since the Raman Rabi rate is small compared with the bandgap.

There are several potential problems with our approach to creating a phase-space distribution. First, violations of adiabaticity and heating from scattering of lattice light will modify the distribution of atoms in the energy eigenstates. Also, Supplementary Equation 1 assumes that the principal axes of the trap are aligned with the lattice directions; a solution for the eigenstates without this constraint is unknown. In our experiment, these axes are not aligned, however. Finally, we assume that the trapping potential is spherically symmetric. In our experiment, the ratio of trap frequencies is 2.4:2.1:1. As the lattice is turned on, the overall trap potential becomes more spherically symmetric. Because the gas is collisionless, cross-dimensional thermalization is not possible [3], and the aspect ratio of the gas remains unchanged as the lattice is superimposed on the gas. We have verified this behavior for the images used to create Supplementary Figure 3. The two principal axes of the trap with trap frequencies in a 2.4:1 ratio lie within the imaging plane. We observe that the density profile of the gas before turning on the lattice has an aspect ratio of approximately 2 along these directions. This aspect ratio remains unchanged at $s = 4$, even though the ratio of trap frequencies along the imaging directions is 1.6:1. The good agreement apparent in Supplementary Figure 3 suggests that all of these complications are minor effects.

SUPPLEMENTARY NOTE 2: TRANSPORT LIFETIME CALCULATION

We use a standard technique to calculate the transport lifetime rate based on Fermi's Golden Rule (FGR) that closely follows the approach used in Refs. 4 and 5. We allow for no free parameters in the calculation of the relaxation rate—experimentally measured values are used for all parameters. We use FGR to calculate the rate at which the Hubbard interaction term $H_I = U \sum_i n_{i,\downarrow} n_{i,\uparrow}$ scatters particles between states of different quasimomentum. In this method, H_I is treated as a perturbation to the tight binding Hamiltonian $-t \sum_{\langle ij \rangle,\sigma} \left(\hat{c}^{\dagger}_{i\sigma} \hat{c}_{j\sigma} + h.c. \right)$. This approximation is only valid in the weakly interacting limit and is not satisfied in the experiment.

For this calculation we treat the $|\uparrow\rangle$ gas as stationary (that is, possessing no net quasimomentum). We use FGR to compute the time rate of change of the total quasimo-

mentum of the $|\downarrow\rangle$ component $\left\langle\!\left\langle \partial\left(\vec{q}_\downarrow\cdot\hat{\delta k}\right)/\partial t\right\rangle\!\right\rangle$ along the direction of $\hat{\delta k}$, where $\langle\!\langle\rangle\!\rangle$ represents a thermodynamic sum over all possible scattering events, and $\hat{\delta k}$ is a unit vector along the Raman wavevector difference. We assume that the total quasimomentum $\left\langle \vec{q}_\downarrow\cdot\hat{\delta k}\right\rangle = \int d^3\vec{r}\int d^3\vec{q}\;\rho_\downarrow\left(\vec{r},\vec{q}\right)\vec{q}\cdot\hat{\delta q}/h^3$ of the $|\downarrow\rangle$ gas decays exponentially, so that the transport lifetime is $\frac{1}{\tau_t} = -\frac{1}{\langle\vec{q}_\downarrow\cdot\hat{\delta k}\rangle}\left\langle\!\!\left\langle \frac{\partial(\vec{q}_\downarrow\cdot\hat{\delta k})}{\partial t}\right\rangle\!\!\right\rangle$.

We use dimensionless spatial and quasimomentum coordinates $\vec{\mathcal{R}} = \vec{r}/d$ and $\vec{\mathcal{Q}} = \pi\vec{q}/q_{\rm B}$, respectively, to carry out this calculation, such that $\frac{1}{\tau_t} = -\frac{1}{\langle\vec{\mathcal{Q}}_\downarrow\cdot\hat{\delta k}\rangle}\left\langle\!\!\left\langle \frac{\partial(\vec{\mathcal{Q}}_\downarrow\cdot\hat{\delta k})}{\partial t}\right\rangle\!\!\right\rangle$. FGR is used to calculate the time-rate-of-change of momentum as

$$\begin{aligned}\left\langle\!\!\left\langle \frac{\partial\left(\vec{\mathcal{Q}}_\downarrow\cdot\hat{\delta k}\right)}{\partial t}\right\rangle\!\!\right\rangle = \frac{1}{2}\frac{2\pi}{\hbar}\frac{U^2}{2t}\int d^3\vec{\mathcal{R}}\int\frac{d^3\vec{\mathcal{Q}}_{1\downarrow}}{(2\pi)^3}\int\frac{d^3\vec{\mathcal{Q}}_{2\uparrow}}{(2\pi)^3}\int\frac{d^3\vec{\mathcal{Q}}_{3\downarrow}}{(2\pi)^3}\,\tilde{\rho}_\downarrow\left(\vec{\mathcal{Q}}_{1\downarrow},\vec{\mathcal{R}}\right)\tilde{\rho}_\uparrow\left(\vec{\mathcal{Q}}_{2\uparrow},\vec{\mathcal{R}}\right)\times\\ \left[1-\tilde{\rho}_\downarrow\left(\vec{\mathcal{Q}}_{3\downarrow},\vec{\mathcal{R}}\right)\right]\left[1-\tilde{\rho}_\downarrow\left(\vec{\mathcal{Q}}_{4\uparrow},\vec{\mathcal{R}}\right)\right]\left(\vec{\mathcal{Q}}_{3\downarrow}-\vec{\mathcal{Q}}_{4\downarrow}\right)\cdot\hat{\delta k}\times\\ \delta\left[\tilde{\epsilon}\left(\vec{\mathcal{Q}}_{1\downarrow}\right)+\tilde{\epsilon}\left(\vec{\mathcal{Q}}_{2\uparrow}\right)-\tilde{\epsilon}\left(\vec{\mathcal{Q}}_{3\downarrow}\right)-\tilde{\epsilon}\left(\vec{\mathcal{Q}}_{4\uparrow}\right)\right].\end{aligned} \quad (2)$$

Supplementary Equation 2 integrates over scattering events between $|\uparrow\rangle$ and $|\downarrow\rangle$ atoms with initial momenta $\vec{\mathcal{Q}}_{1\downarrow}$ and $\vec{\mathcal{Q}}_{2\uparrow}$ and final momenta $\vec{\mathcal{Q}}_{3\downarrow}$ and $\vec{\mathcal{Q}}_{4\uparrow} = \vec{\mathcal{Q}}_{3\downarrow} - \vec{\mathcal{Q}}_{1\downarrow} - \vec{\mathcal{Q}}_{2\uparrow}$. The value of $\vec{\mathcal{Q}}_{4\uparrow}$ is mapped into the first BZ by subtracting reciprocal lattice vectors if it exceeds the first BZ boundary. The dimensionless tight-binding energies are $\tilde{\epsilon}\left(\vec{\mathcal{Q}}\right) = 2\left(3-\cos\mathcal{Q}_x-\cos\mathcal{Q}_y-\cos\mathcal{Q}_z\right)$.

We evaluate Supplementary Equation 2 using Markov-chain Monte Carlo integration. The energy-conserving delta function is represented as a decaying exponential proportional to $e^{-|\tilde{\epsilon}|/l}$. We make l sufficiently small such that the error introduced by representing the delta function this way is smaller than the Monte Carlo integration uncertainty, which is approximately 1%. For Fig. 5 in the Methods section, we use the value of $\tilde{\mu}$ and $\tilde{T}$ averaged across the data to generate the theory curve, while for Fig. 3 in the main text, we use the average value of N.

SUPPLEMENTARY NOTE 3: DMFT CALCULATION

Dynamical mean field theory maps a many-body lattice model to a local single-site problem, where the influence from adjacent sites is replaced by an effective coupling to an external bath [6]. We adapted the code provided in the Toolbox for Research on Interacting Quantum Systems (TRIQS) [7], which employs iterated perturbation theory to obtain the Green's

functions for the Hubbard model with a Bethe lattice geometry and particle-hole symmetry. Despite the simplicity of this method, it is believed to give a qualitatively correct result and scalings for physical quantities [6, 8]. The conductivity $\sigma(\omega)$ for a charged system under the bubble-pair approximation can be calculated as [9]:

$$\sigma(\omega) = \frac{\pi e^2}{\hbar} \int_{-\infty}^{\infty} d\epsilon \Psi(\epsilon) \int_{-\infty}^{\infty} d\omega' \frac{f(\omega') - f(\omega + \omega')}{\omega} A(\omega', \epsilon) A(\omega' + \omega, \epsilon), \tag{3}$$

where e is the charge of the carriers, the local spectral function $A(\omega, \epsilon) = -\mathrm{Im}\left(\frac{1}{\omega+\mu-\Sigma(\omega)-\epsilon}\right)/\pi$ (equivalent to $\rho(\omega)$ defined Eq. 230 in Ref. 6), $\Sigma(\omega)$ is the self-energy, μ is the chemical potential, and $f(\omega)$ is the Fermi-Dirac distribution. $\Psi(\epsilon) = \sum_k \left(\frac{\partial \epsilon_k}{\partial k}\right)^2 \delta(\epsilon - \epsilon_k)$ is the transport density of states, and ϵ_k is the energy of the state in the lattice. For the Bethe lattice, $\Psi(\epsilon) = \Psi(0)\left[1 - (\epsilon/W)^2\right]^{3/2}$, where W is the half-bandwidth.

In our DMFT calculation, the temperature and energies are in units of the half-bandwidth W, which is $6t$ for our lattice. Supplementary Figure 4 shows the dependence of the dc resistivity $\rho = 1/\sigma(0)$ (in arbitrary units) on the interaction strength at $T/W = 0.248$, which corresponds to the conditions for Fig. 5 in the Methods section. Within the interaction range that our measurements explored, the resistivity ρ follows a $(U/W)^2$ scaling for $U/W < 1$. This dependence is consistent with Fermi-liquid theory. At higher U, a slight deviation from quadratic scaling is observed. For the range of temperature we sample, although the weight of quasiparticles at the Fermi energy decreases as U increases, a well-resolved peak in the vicinity of the Fermi level still exists (Fig. 4a in the main text), and the weight in the lower and higher Hubbard bands remains negligible.

Supplementary Figure 5 plots the dependence ρ on temperature at $U/W = 0.383$, corresponding to the conditions for Fig. 4b in the main text. At low T ($T/W < 0.1$), the resistivity shows a quadratic scaling of T, which is the prediction from Fermi-liquid theory. As T increases, a linear-dependence on temperature appears, indicating that the picture describing transport as coherent scattering among well-defined quasiparticles is invalid.

Fig. 4 in the main text shows fits to these scaling laws: $\varrho = b_U + g_U\,(U/t)^2$ (Fig. 4a) and $\varrho = b_T + g_T k_\mathrm{B} \tilde{T}/t$ (Fig. 4b) with offsets b_U, b_T and proportionality constants g_U, g_T as free parameters, which cannot be determined from DMFT. An offset is necessary for the interaction dependence because the Hubbard approximation and tight-binding limit fail in the $s \to 0$ limit. For the temperature scaling, the cross-over to the Fermi-liquid T^2 scaling

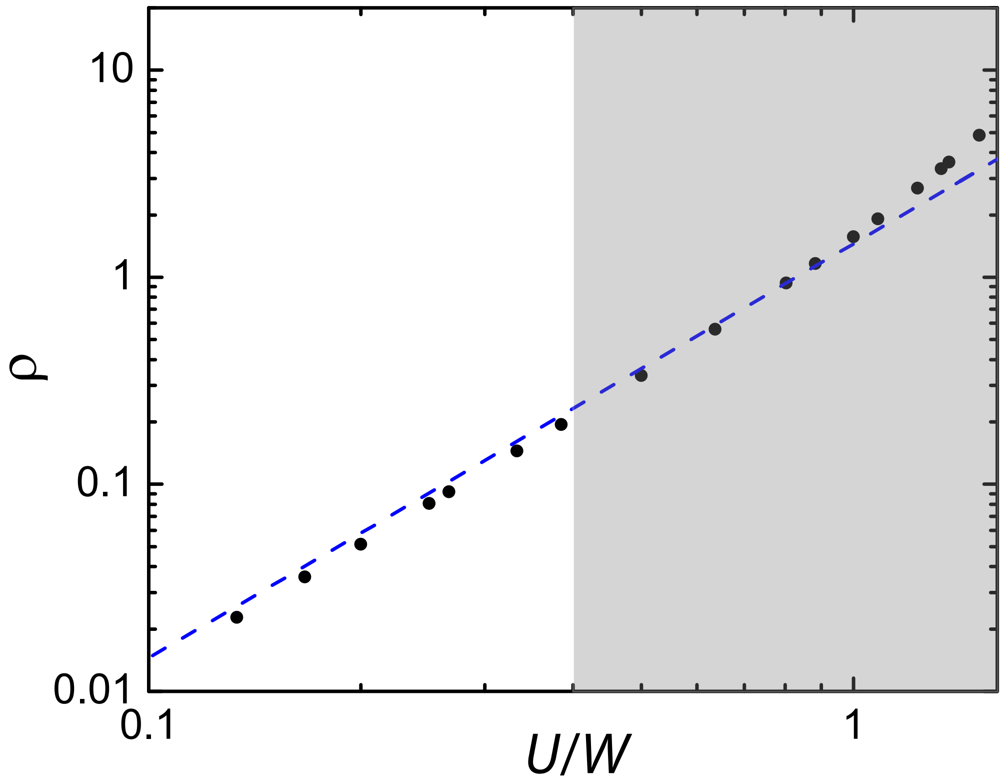

Supplementary Figure 4. DMFT prediction for scaling of resistivity with interaction. The dependence of the resistivity on interaction strength is shown for $k_{\mathrm{B}}T/W = 0.248$, which corresponds to the averaged temperature for the relaxation time measurements shown in Fig.3 of the main text. The blue dashed line is a fit to $(U/W)^2$ for $U/W < 1$. The shaded region shows the area of this plot relevant to the experiment, which samples $U/W = 0.4$–1.5.

at low temperature requires an offset for fitting to T-linear behavior at high T.

Our DMFT calculation assumes particle-hole symmetry and half-filling. The experiment, however, involves an unequal number of $|\downarrow\rangle$ and $|\uparrow\rangle$ atoms and an inhomogeneous density profile. While the particle-hole asymmetry is known to modify the spectral function $A(\omega)$ [10] and affect the absolute value of resistivity, more sophisticated DMFT calculations indicate that T-linear resistivity still appears within the temperature range we sample [11]. Other methods, such as quantum Monte Carlo calculations [12], also show the existence of T-linear scaling within a wide range of filling factors. As we discuss in the Methods section, the density dependence is expected to be a weak effect for the range of temperatures we sample.

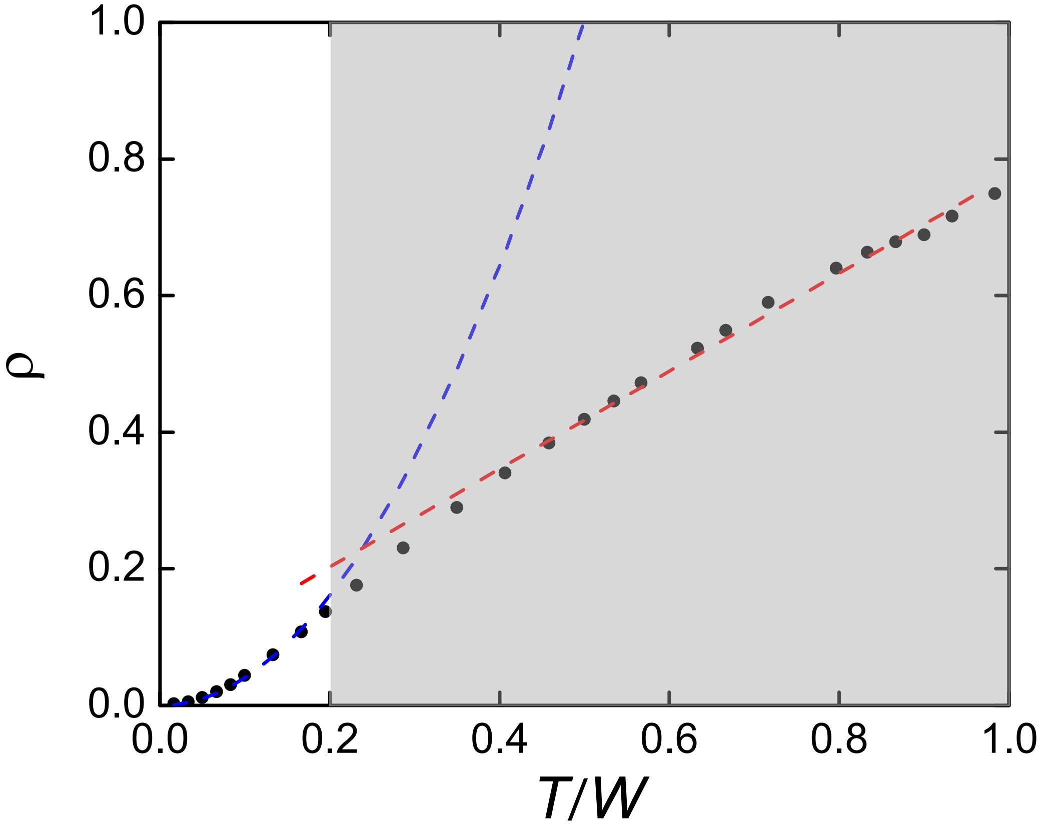

Supplementary Figure 5. DMFT prediction for scaling of resistivity with temperature. The temperature dependence of resistivity is shown at $t/W = 0.383$, corresponding to a lattice depth $s = 4E_{\mathrm{R}}$. At very low temperature, the resistivity is proportional to T^2 as expected in Fermi-liquid theory, suggesting coherent scattering between well-defined quasiparticles. As T increasing, a linear dependence on T appears, corresponds to the bad metal regime of transport. The blue dashed line is a fit to a quadratic function for $T/W < 0.1$, and the red dashed line is a linear fit for $T/W > 0.1$. The shaded region shows the area of this plot relevant to the experiment, which samples $T/W = 0.2$–1.3.

SUPPLEMENTARY NOTE 4: DENSITY-WEIGHTED DENSITY

The density-weighted density inferred from the atom number, trap frequency, and effective temperature is shown in Supplementary Figure 6.

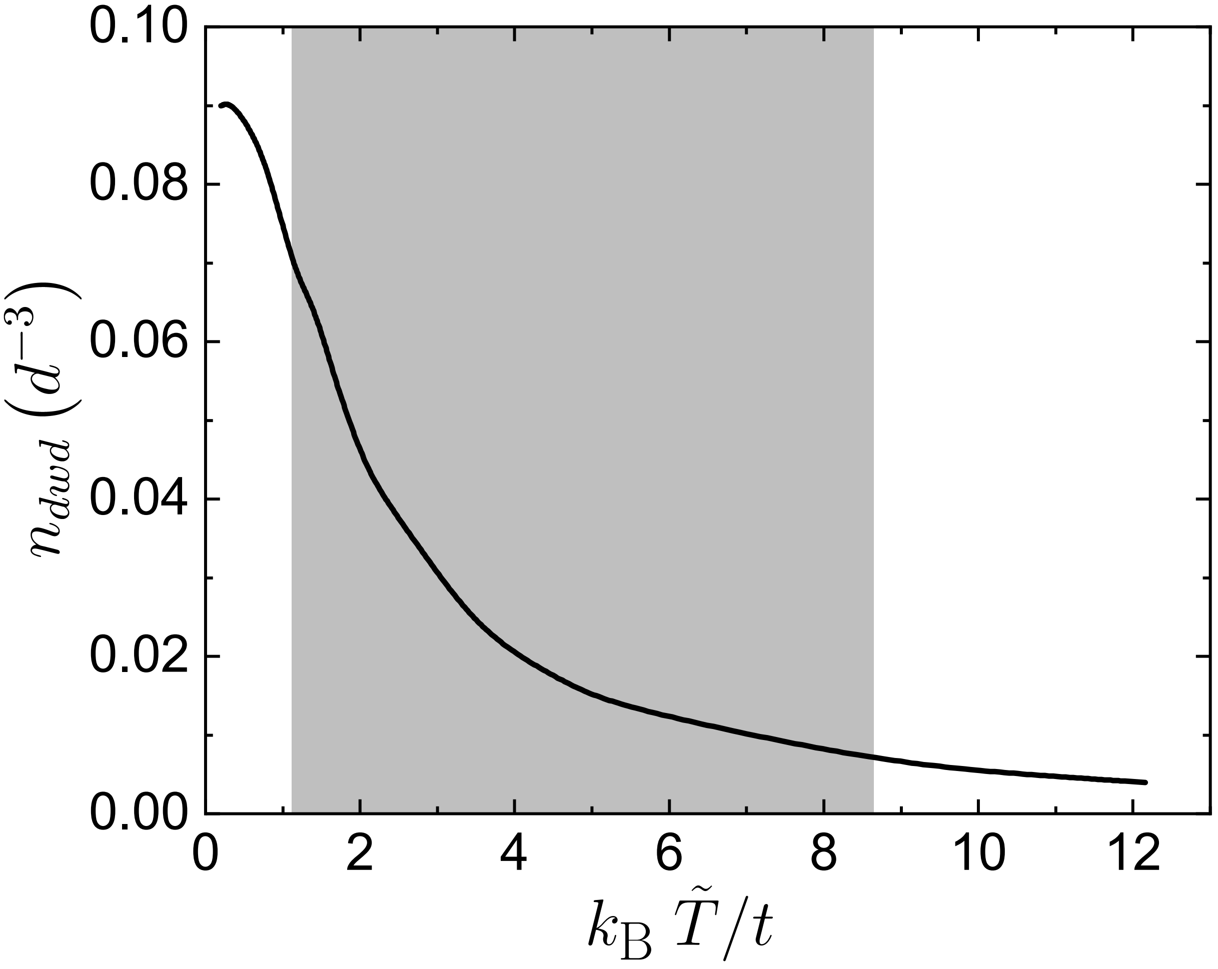

Supplementary Figure 6. Density-weighted density scaling with temperature. The density-weighted density is shown in units of the inverse unit cell volume for varied effective temperature. The shaded region is accessed by the experiment.

SUPPLEMENTARY NOTE 5: LINEAR RESPONSE

Our analysis and solution to the Boltzmann equation assumes linear damping, which is the equivalent of ohmic response. We examine the residuals from the fits used to determine τ_t to check for non-linear response. If non-linearity is present, the residuals will show systematic shifts (from zero) that vary with hold-time. Sample residuals are shown across the full range of data in Supplementary Figure 7 for data taken at $U/t = 2.3$ (corresponding to the data in Figs. 3 and 4b in the main text) and in Supplementary Figure 8 for data taken at low temperature and varied s (corresponding to the data in Fig. 4a in the main text). The uniform scatter around zero and absence of systematic shifts with t_{hold} indicate that

non-linear effects are minor.

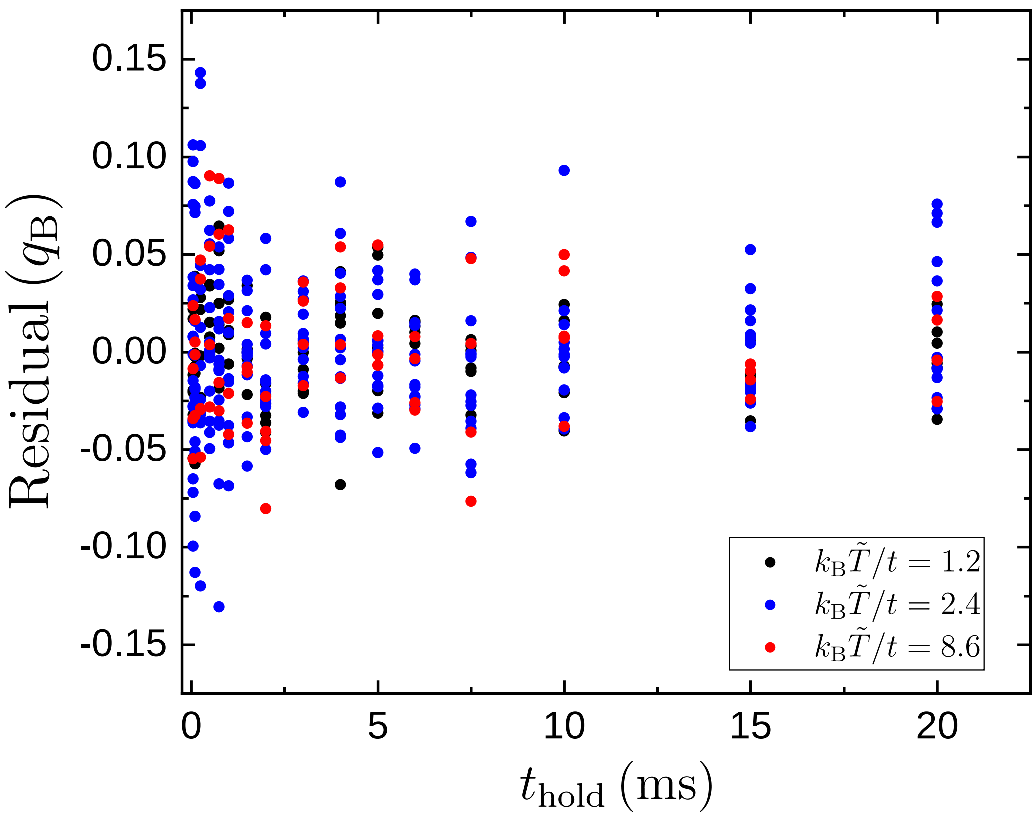

Supplementary Figure 7. Sample residuals for the fits used to determine τ_t. These data were taken with $U/t = 2.3$. The lack of systematic structure in the residuals suggests that the linear response assumption used for the fit model is a reasonable approximation to the relaxation process.

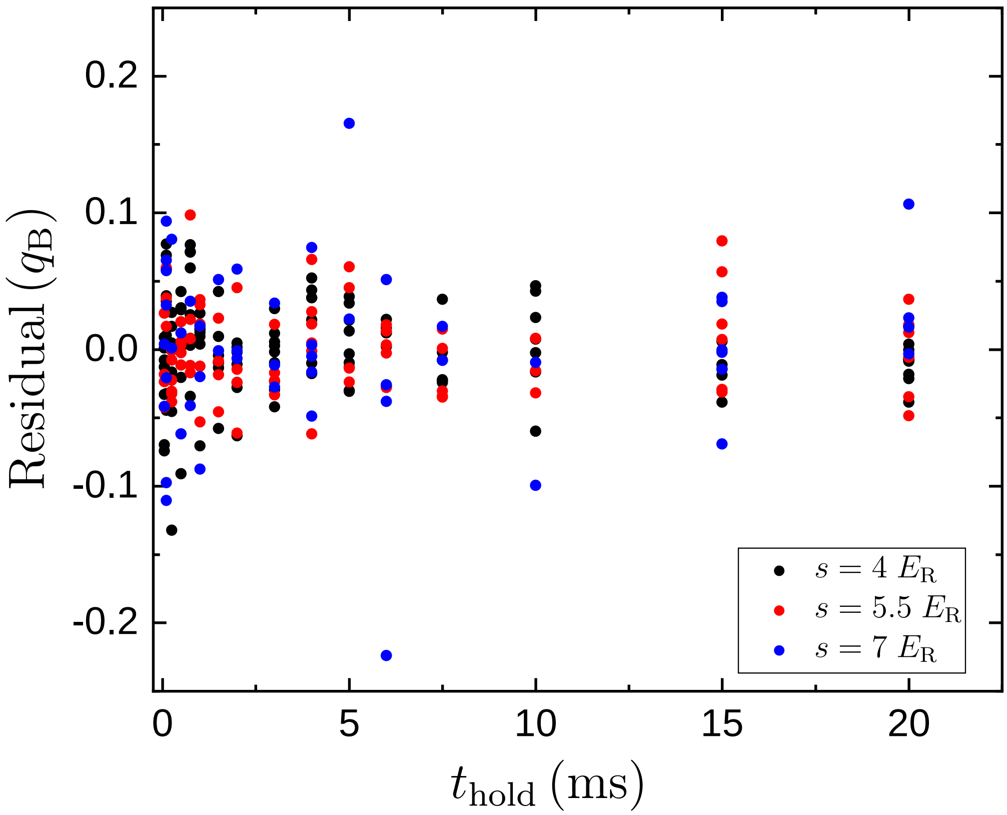

Supplementary Figure 8. Sample residuals for the fits used to determine τ_t. These data were taken with $T/T_F \approx 0.25$. The absence of systematic deviations in the residuals supports the linear response assumption used for the fit model.

SUPPLEMENTARY REFERENCES

[1] Rey, A. M., Pupillo, G., Clark, C. W. & Williams, C. J. Ultracold atoms confined in an optical lattice plus parabolic potential: A closed-form approach. *Phys. Rev. A* **72**, 033616 (2005).

[2] McKay, D., White, M. & DeMarco, B. Lattice thermodynamics for ultracold atoms. *Phys. Rev. A* **79**, 063605 (2009).

[3] DeMarco, B., Bohn, J. L., Burke, J. P., Holland, M. & Jin, D. S. Measurement of p-wave threshold law using evaporatively cooled fermionic atoms. *Phys. Rev. Lett.* **82**, 4208–4211 (1999).

[4] Chen, D., Meldgin, C. & DeMarco, B. Bath-induced band decay of a Hubbard lattice gas. *Phys. Rev. A* **90**, 013602 (2014).

[5] McKay, D. C., Meldgin, C., Chen, D. & DeMarco, B. Slow thermalization between a lattice and free Bose gas. *Phys. Rev. Lett.* **111**, 063002 (2013).

[6] Georges, A., Kotliar, G., Krauth, W. & Rozenberg, M. J. Dynamical mean-field theory of strongly correlated fermion systems and the limit of infinite dimensions. *Rev. Mod. Phys.* **68**, 13–125 (1996).

[7] Parcollet, O., Ferrero, M., Ayral, T., Hafermann, H., Krivenko, I., Messio, L. & Seth, P. TRIQS: A toolbox for research on interacting quantum systems. *Comput. Phys. Commun.* **196**, 398–415 (2015).

[8] Georges, A. & Kotliar, G. Hubbard model in infinite dimensions. *Phys. Rev. B* **45**, 6479–6483 (1992).

[9] Pakhira, N. & McKenzie, R. H. Absence of a quantum limit to charge diffusion in bad metals. *Phys. Rev. B* **91**, 075124 (2015).

[10] Kajueter, H. & Kotliar, G. New iterative perturbation scheme for lattice models with arbitrary filling. *Phys. Rev. Lett.* **77**, 131–134 (1996).

[11] Deng, X., Mravlje, J., Žitko, R., Ferrero, M., Kotliar, G. & Georges, A. How bad metals turn good: Spectroscopic signatures of resilient quasiparticles. *Phys. Rev. Lett.* **110**, 086401 (2013).

[12] Jarrell, M. & Pruschke, Th. Anomalous properties of the Hubbard model in infinite dimensions. *Phys. Rev. B* **49**, 1458–1461 (1994).
